# Multi‐jet fusion for additive manufacturing of radiotherapy immobilization devices: Effects of color, thickness, and orientation on surface dose and tensile strength

**DOI:** 10.1002/acm2.13548

**Published:** 2022-02-25

**Authors:** Amirhossein Asfia, Basaula Deepak, James Ivan Novak, Bernard Rolfe, Tomas Kron

**Affiliations:** ^1^ School of Engineering Faculty of Science, Engineering and Built Environment Deakin University Victoria Australia; ^2^ ARC Industrial Transformation Training Centre in Additive Bio‐manufacturing Queensland University of Technology Queensland Australia; ^3^ Department of Physical Science Peter MacCallum Cancer Centre Victoria Australia; ^4^ School of Architecture, Faculty of Engineering, Architecture and Information Technology The University of Queensland Queensland Australia; ^5^ Herston Biofabrication Institute Metro North Hospital and Health Service Level 12, Block 7, Royal Brisbane and Women's Hospital Herston Queensland Australia

**Keywords:** 3D printing, cancer treatment, dosimetric testing, HP jet fusion 580, medical device manufacture, multi‐color, powder bed fusion

## Abstract

Immobilization devices are used to obtain reproducible patient setup during radiotherapy treatment, improving accuracy, and reducing damage to surrounding healthy tissue. Additive manufacturing is emerging as a viable method for manufacturing and personalizing such devices. The goal of this study was to investigate the dosimetric and mechanical properties of a recent additive technology called multi‐jet fusion (MJF) for radiotherapy applications, including the ability for this process to produce full color parts. Skin dose testing included 50 samples with dimensions 100 mm × 100 mm with five different thicknesses (1 mm, 2 mm, 3 mm, 4 mm, and 5 mm) and grouped into colored (cyan, magenta, yellow, and black (CMYK) additives) and non‐colored (white) samples. Results using a 6 MV beam found that surface dose readings were predominantly independent of the colored additives. However, for an 18 MV beam, the additives affected the surface dose, with black recording significantly lower surface dose readings compare to other colors. The accompanying tensile testing of 175 samples designed to ASTM D638 type I standards found that the black agent resulted in the lowest ultimate tensile strength (UTS) for each thickness of 1–5 mm. It was also found that the print orientation had influence on the skin dose and mechanical properties of the samples. When all data were combined and analyzed using a multiple‐criteria decision‐making technique, magenta was found to offer the best balance between high UTS and low surface dose across different thicknesses and orientations, making it an optimal choice for immobilization devices. This is the first study to consider the use of color MJF for radiotherapy immobilization devices, and suggests that color additives can affect both dosimetry and mechanical performance. This is important as industrial additive technologies like MJF become increasingly adopted in the health and medical sectors.

## 1 INTRODUCTION

Radiotherapy is one of the most effective techniques for the cancer treatment, but does come with the risk of damaging healthy cells surrounding the treatment site.^1^, ^2^ In order to reduce the toxic side effects of radiation therapy, immobilization devices are used to hold the patient firmly on the treatment couch and minimize exposure of healthy cells to high‐energy radiation beams by keeping the patients in the correct position during the course of treatment.^3^ However, these devices can have a bolus effect that increases the radiation dose on the patients’ skin;^4^, ^5^, ^6^ consequently, surface dose measurement is essential to measure the radiation dose on the patient's skin during the course of the treatment.^7^ Significant increase in the radiation dose through build‐up effect may lead to painful reddening of the surface tissue.^8^

Increasingly, new technologies are being utilized in the production of immobilization devices to improve fit and patient comfort, thereby increasing the accuracy of treatment compared to traditional methods such as the use of thermoplastic sheets manually formed around patient anatomy. Additive manufacturing (AM), also known as 3D printing, has shown promising results in a growing body of research studies due to the ability to directly manufacture accurate and personalized immobilization devices from 3D patient geometry captured through medical imaging or 3D scanning methods.^3^ Research has included the development of a 3D printed treatment shell to immobilize a patient's head and neck,^9^ evaluating the radiation dose change in the 3D print materials and finding that all the materials (VisiJet Clear, EOS PA3200 GF, Object ‘VeroWhite’) increased the skin dose. Kohli et al.^10^ investigated the effect of thin < 0.5 mm layers of paint on immobilization devices, finding a negligible influence on the surface dose. However, as novel AM technologies allow color to be directly printed within the material as part of the production process, rather than painted on the exterior, new research is needed to understand if the additives affect the surface dose.

As well as consideration of dosimetry, additively manufactured immobilization devices need to be mechanically strong. One of the challenges with AM is the layer‐by‐layer fabrication process which can result in weakness between layers, and different AM technologies will result in different strength properties. For example, a recent study linked tensile testing of Fused Disposition Modeling (FDM) samples with surface dose testing, finding that samples with maximal ultimate tensile strength (UTS) often recorded a high surface dose,^4^ while weak samples likely to be unsuitable for immobilization recorded a low surface dose. Balance is needed between strength and surface dose, with the researchers finding that the stars infill pattern resulted in an optimal strength with low surface dose across different print orientations. However, printing technologies such as multi‐jet fusion (MJF) do not utilize infill geometries due to the way they build parts within a bed of powder, resulting in solid material sections unless some internal geometry is specifically designed as part of the 3D file. Pandzic et al.^11^ investigated the effect of color on a FDM 3D print through tensile testing, confirming that color can affect the UTS. UTS varied from 35 MPa to 46 MPa depending on the sample's color. The red samples had the maximum UTS of 46 MPa and the pink samples had the minimum UTS of 35 MPa.

While FDM is a well‐established AM technology of interest in radiotherapy, newer technologies are emerging that provide different functional and aesthetic opportunities. MJF is a recent powder‐based AM technique developed by Hewlett‐Packard (HP) that is reportedly 10 times faster than FDM print technology.^12^ MJF is an industrial AM process capable of manufacturing functional prototypes made of nylon and end‐use production products in large quantities. The name of the MJF technology comes from the multiple inkjet heads, similar to the inkjet heads of conventional print technology, which performs the manufacturing process. The inkjet head arrays move across the print bed to perform agent distribution, material recoating, and heating. Depending on the specific MJF hardware, this process can include the deposition of color agents using cyan, magenta, yellow, and black (CMYK) ink cartridges, allowing full‐color 3D printing as the CMYK agents are blended.^13^ These inks are made up of proprietary additives.

Similar to selective laser sintering (SLS), MJF uses an enclosed heated chamber where powdered polymer, typically polyamide 11 (PA11) or polyamide 12 (PA12), is spread across a build plate. However, rather than a laser, a fusing agent is deposited by the print heads across the powder, where the cross‐section of a part is required. An additional detailing agent forms the perimeter of the part, before final fusion of the powder particles is caused by an infrared (IR) lamp as the heat source.^14^ Where full‐color printing is possible, a white detailing agent is deposited around the perimeter before CMYK ink features are added to finalize the full‐color parts. This leads to a cross‐section comprised of an internal region (black) and external (white or colored) surface. Finally, a new layer of powder is spread over the top and the process repeats. The layer thickness for a HP printer such as HP MJF 580 is 0.08 mm and the effective building volume or the maximum part size is 332×190×248 mm. The HP machine includes built‐in capability to remove much of the unused powder and re‐use it later, before allowing an operator to open it and further vacuum loose powder away. Bead blasting is then commonly used to remove all excess powder, resulting in the finished parts which may be further dyed or clear‐coated. This system has been attractive for many industries including medical applications, for example, the production of molds to create clear dental aligners,^15^ although the materials themselves have not been classified as biocompatible at the time of writing.

Researchers investigating the mechanical performance of MJF parts found that samples oriented at a 45° angle to the build platform had the strongest tensile performance compared to horizontal or vertical.^16^ O’ Conner et al.^17^ investigated the mechanical properties of reinforced glass bead PA12 and PA12 manufactured using MJF print technology, finding a reduced flexural and tensile strength by the addition of glass beads. O'Conner et al.^18^ also evaluated the mechanical properties of 3D printed PA12 parts manufactured using MJF technology. Their work highlighted the isotropic behavior of MJF parts with regard to tensile strength, with a recorded maximum tensile strength of 49 MPa. However, for flexural testing the results confirmed the significant influence of the print orientation on the flexural strength of the printed samples; compared to a horizontal orientation, the vertical orientation had a 40% higher flexural strength.

As a technology capable of high strength parts and fine details, as well as a machine capable of relatively fast production, this may have many applications within clinical contexts, for example, radiotherapy immobilization devices. In order to validate MJF for this application, this research adopted an established methodology^4^ to compare the effect of print orientation and thickness on the mechanical and dosimetry properties of samples. Additionally, different colored samples were used to assess whether the color had any influence on the dosimetric and mechanical properties of the printed samples, and identify the optimal color. This provides clinicians and engineers with new guidelines for utilizing MJF AM compared to the more established FDM guidelines.

## 2 METHODS

In order to define the optimum combination of print orientation and different additives (i.e., colors) to produce a fixation device for the radiation therapy, two separate tests were conducted before combining the data: first surface dose measurements were conducted with 50 samples, and then tensile strength was measured using 175 samples. Print orientation has influence on print speed and quality. For instance, by changing the print orientation, the dosimetric and mechanical properties of the samples will change. Hence, we have investigated the influence of different print orientations and additives on the dosimetry and mechanical properties of the samples.

### 2.1 Process parameters and 3D printing

#### 2.1.1 Manufacturing of samples for surface dose testing

Square samples measuring 100 × 100 mm were designed using computer‐aided design (CAD) software Fusion 360 (Autodesk, Marin, USA). Five thicknesses (1 mm, 2 mm, 3 mm, 4 mm, and 5 mm) were chosen, and five additives for coating matching the inks of the HP Jet Fusion 580 3D printer (cyan, magenta, yellow, black, and white). Models were exported to Stereolithography (STL) format. STL files were then imported into Netfabb (Autodesk, Marin, USA) and used to arrange the STL files in orientation and quantity listed below:

- 1 of each thickness (1 mm, 2 mm, 3 mm, 4 mm, and 5 mm) with no added additives (white) at two orientations (45° and 90°) = 10 samples.
- 1 of each thickness (1 mm, 2 mm, 3 mm, 4 mm, and 5 mm) in each additive (cyan, magenta, yellow, black, and white) at 0° orientation = 25 samples.
- 5 identical samples with 3 mm thickness and with white additive for investigating the reproducibility = 5 samples.

This resulted in 40 total samples. The parts were sliced and converted to G‐code in HP SmartStream 3D Build Manager software (HP, Palo Alto, USA). G‐code is the low‐level machine language that controls the printer.^19^ Polyamide 12 (PA12) was used as the print material, with properties detailed in Table 1. The process parameters are also detailed in Table 1.

**TABLE 1:** HP Jet Fusion 580 process parameters and PA12 powder specifications

Process parameters		
Property	**Value**	**Normative**
Effective build volume	332×190×248 mm	NA
Layer thickness	0.08 mm	NA
Build speed	1817 cm^3^/h	NA
Print head resolution	1200 dpi	NA

PA12 Powder specifications		
Powder melting point (DSC)	187^°^C	ASTM D3418
Particle size	60 μm	ASTM D3451
Bulk density of powder	0.425 g/cm^3^	ASTM D1895

#### 2.1.2 Manufacturing of samples for computed tomography number and density measurement

Twenty‐seven 30 × 30 mm^2^ samples with a 5 mm thickness were designed using Fusion 360 CAD software. The models were then saved in STL format and converted to G‐code in HP SmartStream 3D Build Manager software. Again, PA12 was used for printing the samples. The samples were printed using an HP Jet Fusion 580 printer in three batches on three different days. Each batch of samples contained five black samples with different print orientations (0°, 20°, 45°, 70°, 90°) and four samples with different additives for coating (white, yellow, magenta, cyan) at 0° print orientation.

#### 2.1.3 Manufacture of samples for tensile testing

Tensile samples were designed in Fusion 360 software based on the ASTM D638 Type I standard, saved in STL format and sent to Netfabb software, where they were organized in quantity and orientation within the 3D printer's build volume. In HP SmartStream 3D Build Manager software, the parts were sliced and converted to G‐code. Similar to the surface dose samples, a combination of thickness, orientation and additives was used as detailed below:

- 5 of each thickness (1 mm, 2 mm, 3 mm, 4 mm, and 5 mm) with no added additive (white) at two orientations (45° and 90°) = 50 samples.
- 5 of each thickness (1 mm, 2 mm, 3 mm, 4 mm, and 5 mm) in each additive (cyan, magenta, yellow, black, and white) at 0° orientation = 125 samples.

In total, 175 samples were printed using the process parameters shown in Table 1 and PA12 material.

### 2.2 Surface dose testing

**FIGURE 1:**
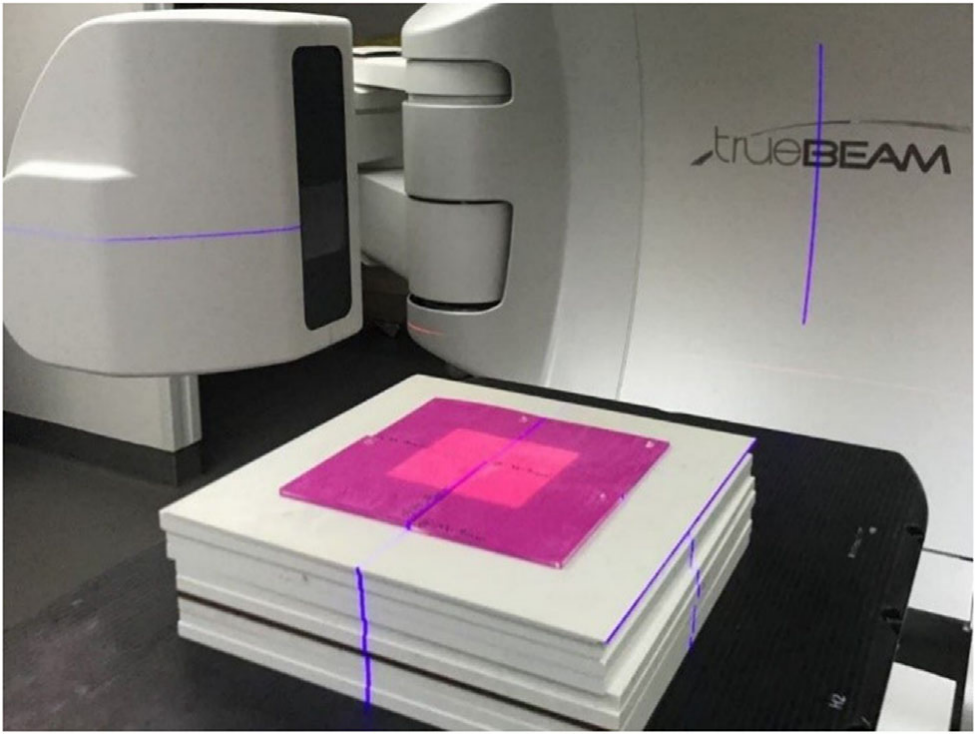
Performing skin dose measurement on a Varian Trubeam linear accelerator on samples of cyan, magenta, yellow, black, and white color and four different thicknesses

Gafchromic EBT3 film (Ashland ISP, Wayne, USA) was used for surface dose measurements due to its excellent spatial resolution, near tissue equivalent, and energy independent dose response.^20^ A Varian Trubeam linear accelerator (Varian Medical Systems, Palo Alto, USA) with 6 MV and 18 MV photon beam energy was used to assess the surface dose from the 3D printed samples. All measurements were performed on RW3 solid water phantom (PTW Freiburg, Freiburg, Germany). Films were cut into ∼30 mm × 30 mm for calibration and ∼100 mm × 100 mm for surface dose measurements noting their orientation. Extreme care was followed to minimize potential measurement uncertainties while handling film; gloves were used to avoid any potential mark on the film which may increase or decrease net optical density of the exposed film. The film pieces were positioned at the surface and a depth of maximum dose (*D*_max_) of 6 MV and 18 MV beam between the solid water slabs. For all exposures, a field of 100 mm × 100 mm and source‐to‐surface distance (SSD) of 1000 mm to the RW3 solid water phantom was used. To create a dose response curve, dose ranges from 0–3000 cGy (0, 20, 50, 100, 300, 500, 700, 1200, 1800, 2400, and 3000) were delivered. 500 cGy dose was delivered to the measurement film pieces. Four samples described in Section 2.1.1 could be tested on each piece of film as shown in Figure 1.

All films were readout on an EPSON Perfection V700 Photo flatbed scanner (Epson, Suwa, Japan). The irradiated films were scanned with 75 DPI scanner resolution with auto exposure and color correction turned off. The central area of the scanner was used to avoid any variation or non‐uniformity across the scanner. Consistent film orientation and scanning direction was used for both calibration and measurement film pieces. Time between radiation exposures to film readout was consistent for both calibration film pieces and measurement film pieces.

Dose analysis was performed using Sun Nuclear SNC patient software version 6.6.0.32313 (Sun Nuclear Corporation, Middleton, USA). According to SNC patient software requirements, a dose–response curve was generated from the red channel of 48 bit RGB image. The mean absorbed dose from the central 10 × 10 mm region of each film piece was used to determine the surface dose by taking a ratio of mean doses between surface and a depth of *D*_max_. For computing *p*‐value, the two‐sided multivariable ANOVA (MANOVA) was used. The confidence level of 95 was defined. The error bar is computed for the 3 mm thick white samples with 0° print orientation, because these samples were printed six times. The error bar is typical for all the samples. The excel software (Microsoft, Washington, USA) is used for computing the error bars for the tensile testing results. The 95% confidence level for mean was selected.

### 2.3 Computed tomography number and density measurement

Samples for the computed tomography (CT) number and density measurements were scanned using a Philips CT scanner (Philips, Amsterdam, the Netherlands). The scan parameters of 140 kVp tube potential, 88 mAs tube current and scan time, and 600 mm field of view (FOV) were used. CT number was recorded by drawing a circular region of interest ∼ 10 mm^2^ in the middle of the axial slice. These samples were printed in 3 days in order to investigate the repeatability of the test results. The samples were weighed using a Sartorius (Sartorius, Goettingen, Germany) weighing machine, and the dimensions of the samples were measured using a calliper in three points for each dimension. The average value was selected, and the density of the samples were computed using Equation (1):

(1) $$\mathrm{Density}\;=\frac{\mathrm{Mass}}{\mathrm{Volume}}\;.$$

### 2.4 Tensile testing

In this work, a 50 KN 5969 Instron (Instron, Norwood, USA) machine was used for performing the tensile test on the samples. According to the ASTM D638 standard, the 5 mm min^–1^ extension rate was selected, and the samples’ extension was measured using an extensometer. The achieved data then were graphed for the 175 samples in a spreadsheet for the calculated engineering stress–strain curve. The UTS, which is the maximum stress that a sample can withstand before breaking, was calculated. The error bars are computed for the tensile testing results. The 95% confidence level for mean was selected.

### 2.5 Combining results

The data from skin dose measurements and tensile tests were combined using the multi criteria decision making (MCDM) technique^21^ with a weighted product model (WPM)^22^ used to identify the optimal process parameters (Table 2). WPM is one of the MCDM techniques. In this technique, based on the multiple criteria, the best alternative among multiple alternatives needs to be found. It is important to choose the appropriate print orientations, sample color additive, and thickness when designing an immobilization device because the device needs to keep balance between the minimum skin dose and maximum tensile strength. Immobilization devices need to be durable and withstand the possible loads that could be applied to them during treatment with minimum deflection, while simultaneously not increasing the radiation dose on the patient's skin when a tumor is at depth.

**TABLE 2:** MCDM technique (WPM) for choosing the best sample's additive for coating

	UTS (MPa)					Surface dose in % (6 MV)					Surface dose in % (18 MV))			
Thickness	1 mm	2 mm	3 mm	4 mm	5 mm	1 mm	2 mm	3 mm	4 mm	5 mm	2 mm	3 mm	4 mm	5 mm
Weight	0.071	0.071	0.071	0.071	0.071	0.071	0.071	0.071	0.071	0.071	0.071	0.071	0.071	0.071
0°‐B	15.6	22	24.8	27.9	31.5	40	53	62	71	73	29	37	43	50
0°‐C	19.6	29.4	32.3	33.1	36	38	52	62	69	76	34	40	47	54
0°‐M	23.3	30.8	32.4	35.4	37.3	41	53	62	70	76	33	41	47	52
0°‐Y	22.6	28	31.5	34	38.1	41	53	67	71	76	31	40	44	50
0°‐W	23.4	29.5	32.1	34.2	37.9	42	53	61	70	84	32	40	46	53

B = Black, C = Cyan, M = Magenta, Y = Yellow, W = White.

## 3 RESULTS

### 3.1 Surface dose measurements

**FIGURE 2:**
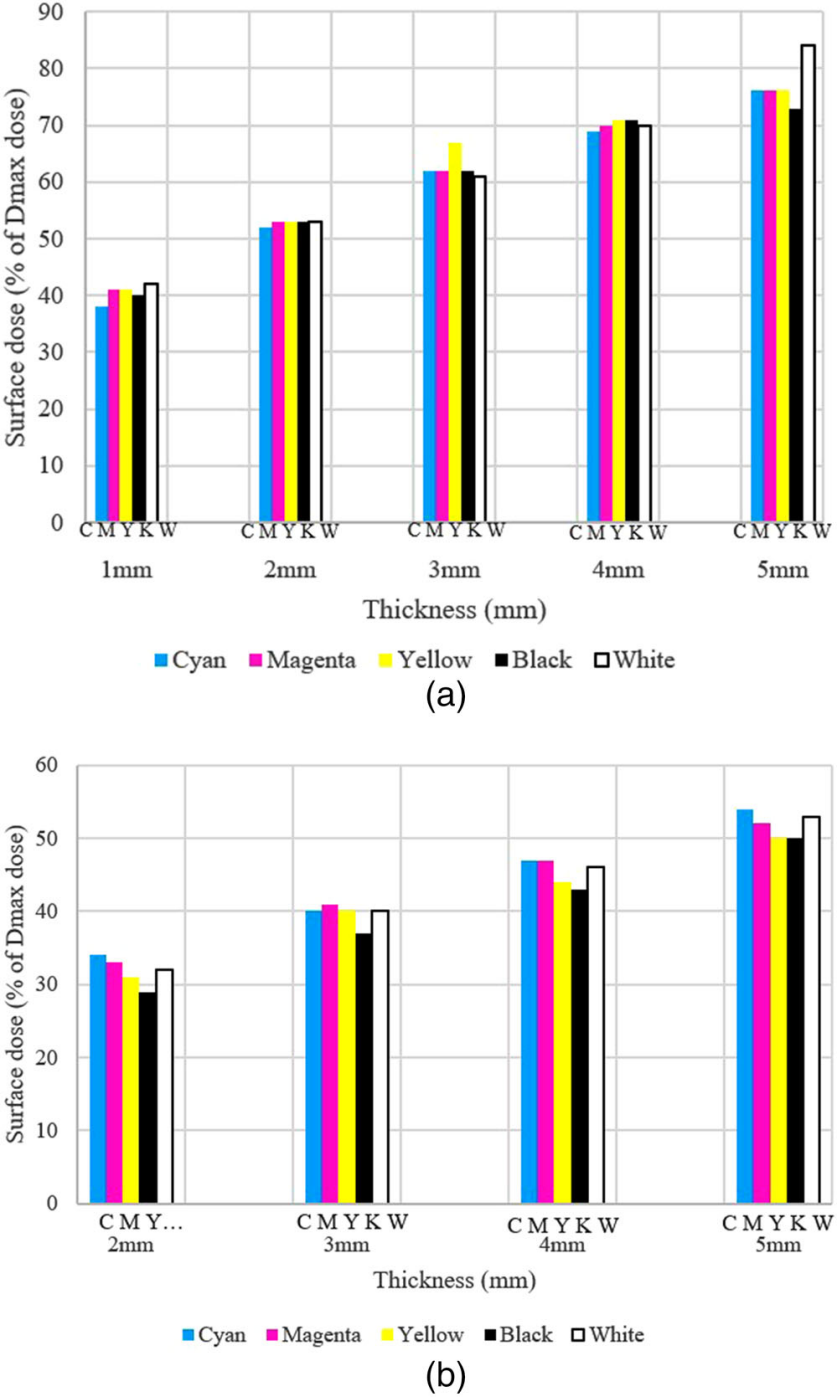
Surface dose measurement of samples with five different additives (colors) for (a) 6 MV and (b) 18 MV beam experiment

The results of the surface dose measurements are elaborated in Table S1 for both the 6 MV and 18 MV beam with different thickness, additives (colors), and orientations of samples. The minimum measured relative surface dose (% of *D*_max_) for the 18 MV beam was 29% (2 mm thick sample, black additive) and the maximum measured dose was 54% (5 mm thick sample, cyan additive). For the 6 MV beam, the minimum relative surface dose was 38% (1 mm thick sample, cyan additive) and the maximum was 84% (5 mm thick sample, white additive). Results confirm that, by increasing the samples’ thickness, the measured surface dose will increase following a second‐order polynomial curve. For the 6 MV beam experiment, results also show that the five additives have similar surface dose results across the range of thickness samples. The *p*‐value computed for the 6 MV beam experiment was 0.313 (*F* = 1.297) which shows the influence of additives on the skin dose was not significant. However, the measured surface dose for 3 mm yellow and 5 mm white samples is slightly higher as shown in Figure 2a.

For the 18 MV beam experiment, results confirm that the black samples had less skin dose compared to the other samples (Figure 2b). Note that it is highly unlikely to use 18 MV beam for modern intensity‐modulated radiation therapy and volumetric modulated arc therapy techniques to treat the head and neck cancer patients thus the measurement of 18 MV may not be relevant in most of clinical scenario. However, for quick comparison to the measurement of 6 MV beam, selected sample thickness (2 mm–5 mm) was measured in 18 MV beam. The calculated *p*‐value for the 18 MV beam was 0.000 (*F* = 17.833) indicating the statistically significant differences between the black samples and the other colored samples for the 18 MV beam experiment. Cyan had the maximum surface dose for 2 mm and 5 mm thick samples, while magenta showed the maximum surface dose for the 3 mm thick samples. For the 4 mm thick samples, magenta and cyan showed a similar skin dose. For the 6 MV beam experiment, results showed that cyan had the minimum surface dose for the majority of samples (1, 2, and 4 mm thick), while white samples recorded the maximum skin dose for the majority of samples (1, 2, and 5 mm thick).

**FIGURE 3:**
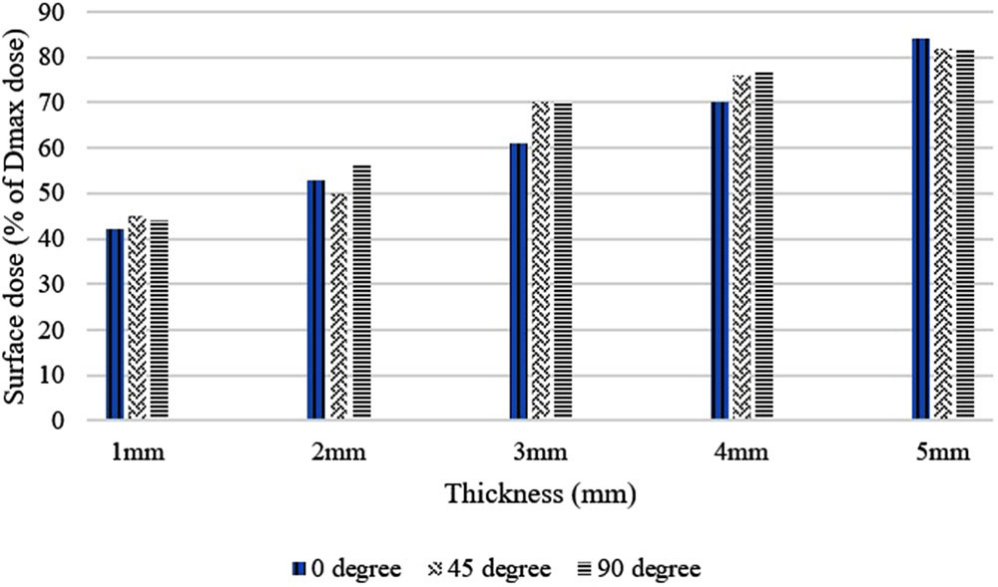
Surface dose measurement of samples with 3 different print orientations for 6 MV beam experiment

The results of the experiments with 0°, 45°, and 90° print orientations and white additive showed that samples printed with 0° print orientation recorded a lower surface dose compared to the samples with 45° and 90° print orientations, except for the 5 mm thick samples (Figure 3). The *p*‐value was computed to investigate the influence of print orientation on the skin dose. The computed *p*‐value was 0.05 which shows that the samples printed with 0° print orientation were statistically different to the other print orientations. Also, five 3 mm thick white samples were irradiated with 6 MV beam to test the reproducibility of print. As is evident in Table S1, the average of five samples measured surface dose is 63%.

### 3.2 Computed tomography number and density measurement

**FIGURE 4:**
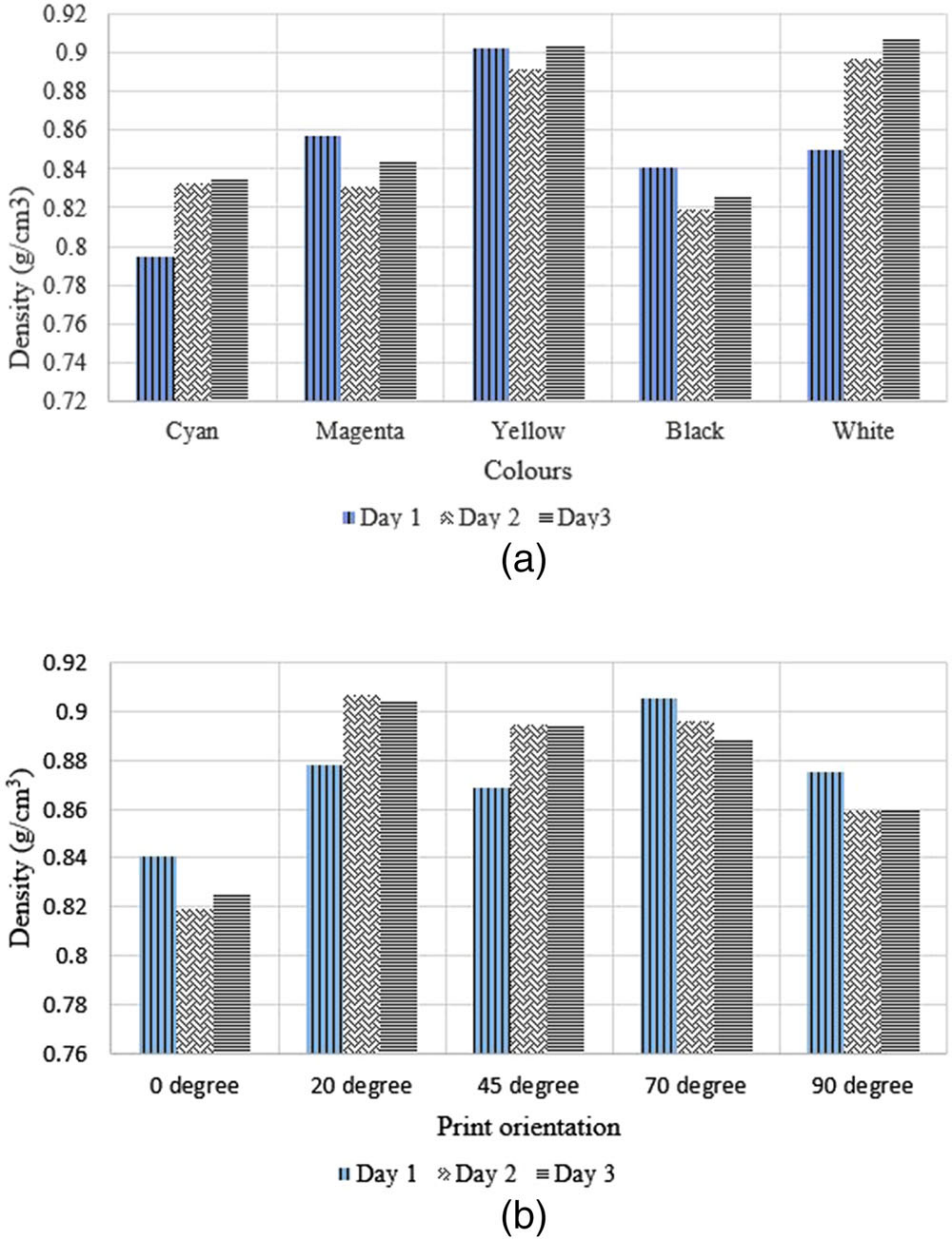
Density measurement of samples with (a) different additives and (b) different print orientations

The results of the scanned samples are shown in Table S2. The *p*‐value was computed to investigate the influence of both print orientation and different color additives on the density which was 0.001 (*F* = 13.569) and 0.004 (*F* = 9.486), respectively. This shows that at least one orientation and one color can be statistically separated from the other classes with respect to sample density. Nevertheless there is variation in the printed results as is evident in Figure 4a, the samples printed with black additive had the lowest density among the samples that were printed on the second and third days, while among the samples that were printed on the first day, the cyan samples had the least density. This may be evidence of variation in the material or print qualities over time or may be due to external factors such as to humidity or another factor that causes variation in density.

As shown in Figure 4b, print orientation also affected the samples’ density. Across all three days, the samples printed with 0° print orientation recorded the lowest density, while the samples with 20° print orientation had the maximum density for the second and third days, with 70° recording the maximum density on day 1. As with the colored samples, there is a reasonable amount of variation in the printed sample density across each printed orientation.

### 3.3 Tensile testing

**FIGURE 5:**
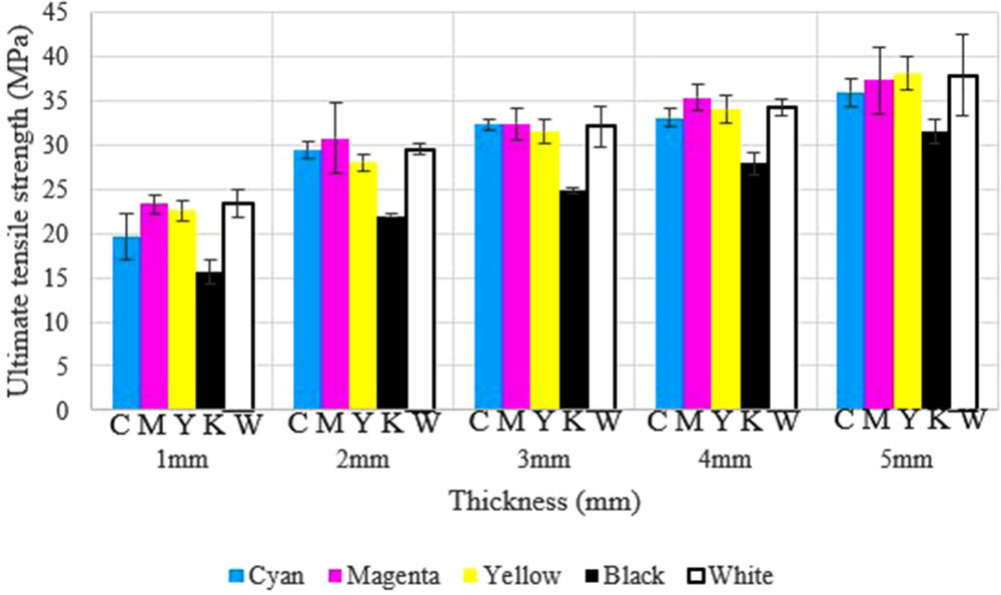
The influence of five different additives on the ultimate tensile strength of the samples

**FIGURE 6:**
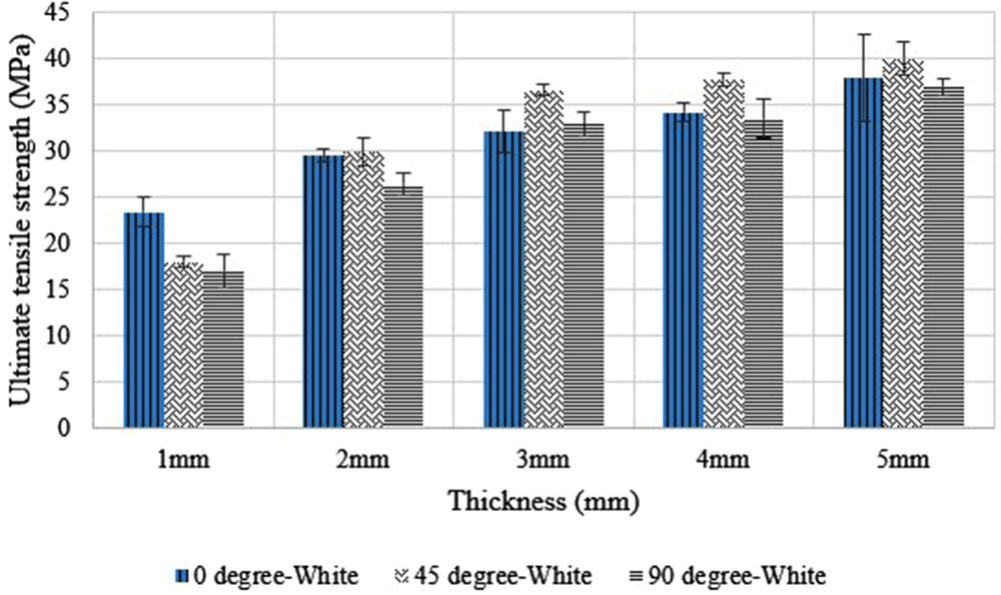
The influence of three different print orientations on the ultimate tensile strength of the samples

The results of the tensile testing are presented in Table S3. The *p*‐value was computed for investigating the effect of different print orientations and different additives on the UTS of the samples. The computed *p*‐values for print orientation and additives were 0.1 (*F* = 3.000) and 15 × 10^–10^ (*F* = 62.114), respectively. This shows that there is a statistically significant difference between the black color additive and the other colors with respect to the UTS of the printed samples. Figure 5 shows that the magenta additive has the highest UTS in the majority of the thicknesses, while the black samples have the lowest UTS. Whereas the print orientation only shows a marginally significant influence on the UTS of the samples. Samples printed with 90° print orientation had the lowest UTS except for the 3 mm thickness (Figure 6).

It should also be noted that the relative standard deviation (RSD) for each thickness of the samples decreased as the thickness of the samples increased. This meant that the thicker samples had closer results than the thinner samples.

### 3.4 Combined mechanical and dosimetry results

#### 3.4.1 Determining the optimum colored additive for radiotherapy immobilization devices

Table 2 shows the combined results of tensile test and surface dose measurement test for the five different CMYKW additives. This table aims at finding the best additive for manufacturing the immobilization device using the HP Jet Fusion 580 additive technology based on the WPM technique. In this technique, the results of tensile test are considered as the beneficial attribute (maximum value desirable) and the results of surface dose tests are considered as the non‐beneficial attribute (lowest value desirable). For the beneficial attribute, the maximum value is identified in each column of the table and each other value in that column will be divided by the maximum value. This way, the UTS results are normalized. On the other hand, the minimum value for the non‐beneficial attributes is computed and the minimum value is divided by each value in that column. After normalizing all the values in the table, we take the power of each normalized component each column with the respective weight of that column (Table 3). Then, the components will be multiplied in each row to calculate the performance score.^22^

**TABLE 3:** Computing the rank of ranks of samples with five different additives by performance score

	UTS (beneficial)					Surface dose (6 MV) (non‐beneficial)					Surface dose (18 MV) (non‐beneficial)				Performance score	Rank
Thickness	1 mm	2 mm	3 mm	4 mm	5 mm	1 mm	2 mm	3 mm	4 mm	5 mm	2 mm	3 mm	4 mm	5 mm	–	–
Weight	0.071	0.071	0.071	0.071	0.071	0.071	0.071	0.071	0.071	0.071	0.071	0.071	0.071	0.071	–	–
0°‐B	0.972	0.976	0.981	0.983	0.987	0.996	0.999	0.999	0.998	1.000	1.000	1.000	1.000	1.000	0.896	5
0°‐C	0.988	0.997	1.000	0.995	0.996	1.000	1.000	0.999	1.000	0.997	0.989	0.994	0.994	0.995	0.944	4
0°‐M	1.000	1.000	1.000	1.000	0.998	0.995	0.999	0.999	0.999	0.997	0.991	0.993	0.994	0.997	0.962	1
0°‐Y	0.998	0.993	0.998	0.997	1.000	0.995	0.999	0.993	0.998	0.997	0.995	0.994	0.998	1.000	0.957	2
0°‐W	1.000	0.997	0.999	0.998	1.000	0.993	0.999	1.000	0.999	0.990	0.993	0.994	0.995	0.996	0.954	3

From these data, the magenta additive was found to have the highest performance score, offering the best balance between a high UTS and low‐surface dose suitable for radiotherapy immobilization devices.

#### 3.4.2 Determining the optimum print orientation

The same MCDM procedure for finding the best print orientation was followed (Table 4). Table 4 represents the combined results of the tensile test and the skin dose measurement test on three different print orientations. This table aims at finding the best print orientation for printing the immobilization device. Table 5 determines the optimal orientation, which is the 0° print orientation because it got the maximum performance score.

**TABLE 4:** MCDM technique (WPM) for choosing the best print orientation

	UTS (MPa)					Surface dose in % (6 MV)				
Thickness	1 mm	2 mm	3 mm	4 mm	5 mm	1 mm	2 mm	3 mm	4 mm	5 mm
Weight	0.1	0.1	0.1	0.1	0.1	0.1	0.1	0.1	0.1	0.1
0°‐W	23.4	29.5	32.1	34.2	37.9	42	53	61	70	84
45°‐W	18	29.9	36.6	37.7	40	45	60	70	76	82
90°‐W	17	26.4	32.9	33.5	37	44	56	70	77	82

**TABLE 5:** Computing the rank of ranks of samples with three different print orientation by performance score

	UTS (beneficial)					Surface dose (6 MV) (non‐beneficial)					Performance score	Rank
Thickness	1 mm	2 mm	3 mm	4 mm	5 mm	1 mm	2 mm	3 mm	4 mm	5 mm	–	–
Weight	0.1	0.1	0.1	0.1	0.1	0.1	0.1	0.1	0.1	0.1	–	–
0°‐W	1.000	0.999	0.987	0.99	0.995	1.000	1.000	1.000	1.000	0.998	0.969	1
45°‐W	0.974	1.000	1.000	1.000	1.000	0.993	0.988	0.986	0.992	1.000	0.935	2
90°‐W	0.968	0.988	0.989	0.988	0.992	0.995	0.994	0.986	0.991	1.000	0.897	3

When combined with the colored additive data, the overall finding from this study was that magenta additive with a 0° print orientation is the optimum parameter for printing immobilization devices.

## 4 DISCUSSION

The use of 3D printing to fabricate personalized immobilization devices is of increasing interest,^3^ and as new AM technologies emerge, there are new opportunities to consider their application to this growing field. MJF technology, as an industrial scale AM process finding value in several medical fields, may offer benefits to clinicians and patients, including the ability to select and blend colors on the exterior surface of a product. However, it is important to first understand the mechanical and dosimetric effects of the colored and non‐colored outcomes, and understand the implications for the design of immobilization devices.

As a general trend, this study identified that surface dose increased as material thickness increased, similar to previous studies that found increases in surface dose as the density of material increased.^4^, ^23^, ^24^ For an immobilization device, this may suggest using the lowest printable thickness is ideal. However, this would ignore the impact material thickness plays on the mechanical strength of a part, which is critical when used to restrict patient movement. Lee et al.^16^ investigated the effect of build orientation on the porosity and mechanical properties of polyamide‐11 manufactured through the MJF technology. Results confirmed that the manufactured parts had a very low porosity of less than 1%. They also found that the samples printed with 45° print orientation exhibited the best tensile mechanical properties in terms of having the highest *E*_Tensile_ ≈ 1500 MPa, σ_Tensile_ ≈ 40 MPa, ε_Break_ ≈ 34%. O'Connor and Dowling^17^ studied the mechanical properties of PA12 and reinforced glass bead PA12. The samples were printed in three print orientations (0°, 90°, and on edge).^25^ All the samples printed in these three print orientations had less than 1% porosity which is less than SLS printed parts which typically shows the porosity between 3%–6%.^25^ This could be due to the combination of planar IR lamps and the fusing agent which is utilized for coating the polymer powder in which upon sintering, it gives a more heterogeneous layer. The results of their study confirm that the samples printed with 90° print orientation had less porosity compared with the other two print orientations.

Unique to the MJF process, the thin exterior layer of colored additives must also be considered from a mechanical and dosimetric perspective. While the specific additives used to create the CMYK coloring are proprietary knowledge, it is clear from this research that they can affect the mechanical and dosimetric performance of parts. While these were not significant for the 6 MV beam, for the 18 MV beam these were sizeable, with surface dose difference up to 5% of maximum dose at the 2 mm thickness between the black (34%) and cyan (29%) colored additives. Such variations can only be attributed to the colored additives, as the internal core and external detailing agent of the printed parts remains the same for each sample. These additives may have different heat absorption characteristics, influencing both the surface dose and mechanical performance of parts. The black color is an obvious example of this, with the color likely to absorb the most heat, which causes a reduced UTS and lower surface dose compared to the colored and white samples. The specific effects of color on the material performance require further investigation to better understand whether the colored dyes themselves play a role in surface dose, or whether they affect the material structure or geometry itself.

The print orientation also influenced the results. Samples that were printed with 0° print orientation exhibited the lowest surface dose and also the lowest density among the other print orientations (45° and 90°). This could be due to the layer‐by‐layer manufacturing manner of the print procedure and the produced porosity in the samples during printing. While previously described as low, the samples with 0° print orientation may have more air gaps inside them and less density which has resulted in reducing the surface dose, as noted in previous studies examining the internal geometry of samples through different print processes.^4^, ^23^, ^24^ However, compared to the parts produced by SLS, the MJF printed parts have higher density and lower porosity.^26^

It is important to note that immobilization devices, including facemasks, have complex structures which cannot be constrained to a specific print orientation. Therefore, design engineers must consider the full UTS data combined with surface dose and make an appropriate selection based on the critical features of a particular immobilization device. The immobilization device needs to reduce the skin dose. It also needs to have optimum mechanical properties, such as high UTS, to withstand the applied load to the device by a patient during the course of treatment. The device needs to have minimum deformation during the treatment when a patient may try to change their head orientation, or when they cough, swallow, or sneeze. Further research may need to quantify the mechanical performance across a broader range of orientations than was possible in this study.

From a human‐centered perspective, the idea of applying colors and graphics to immobilization devices is novel and enabled by color 3D printing technology like MJF. This may be of particular interest in pediatrics, where colors may help ease the anxiety of wearing an immobilization device and create distraction.^10^ If a child were to pick a graphic with predominantly dark/black colors, a designer of an immobilization device may need to consider increasing the thickness in order to compensate for the reduced UTS, while also considering the resulting increase to surface dose. Alternatively, designers may prefer to create a range of graphics of a more magenta hue in order to offer an improved balance between strength and surface dose.

This research is an initial attempt to quantify the performance of colored MJF, and is not exhaustive in its analysis of color. For example, the influence of blending of multiple colors on the skin dose and mechanical properties of the samples was not tested, with only the direct CMYK additives considered that match the cartridges of the HP Jet Fusion 580 process. Additionally, these additives could be investigated for manufacturing boluses and finding the additives that increase the delivery dose of radiation beam on the patient's skin. The number of samples that were used in this study for investigating the influence of additives and print orientations on the skin dose could also be increased to improve the certainty of measurements, however, the cost of producing such a large number of samples must be considered. It is recommended that research must also investigate the influence of different colored additives on other mechanical properties, such as flexural and compressive, for other medical applications such as prostheses, implants, and surgical cutting guides.

## 5 CONCLUSION

This is the first study to examine the application of MJFAM with colored additives for the creation of radiotherapy immobilization devices. It provides new knowledge about the combined effect of part thickness, print orientation, and color on the dosimetric and mechanical performance of samples, finding that the magenta‐colored additive was the overall optimal choice for applications, where high strength and low surface dose was required. However, there were numerous instances, where mechanical or dosimetric performances vary as parts become thicker or are printed in different orientations with different colors, and design engineers must consider the specific geometry of their device in order to optimize it for production with MJF. This research will assist those working on radiotherapy applications of AM, as well as other industries looking to utilize colored MJF to develop products that balance mechanical and aesthetic qualities with color.

## CONFLICT OF INTEREST

The author declares that there is no conflict of interest that could be perceived as prejudicing the impartiality of the research reported.

## AUTHOR CONTRIBUTION

Amirhossein Asfia performed the tensile strength and skin dose measurement experiments, analyzed the data and wrote the paper. Basaula Deepak performed the skin dose measurement test and analyzed the data. James Novak, Bernard Rolfe, and Tomas Kron supervised the work, analyzed the data, designed the experiments, and revised the paper. All authors revised the paper.

## Supporting information



Tables

Supporting Information
