# Supplementary material for: Multi‐jet fusion for additive manufacturing of radiotherapy immobilization devices: Effects of color, thickness, and orientation on surface dose and tensile strength
Source: J Appl Clin Med Phys. 2022 Feb 25;23(4):e13548. doi: 10.1002/acm2.13548 (PMC8992947; doi:10.1002/acm2.13548)
Supplement: Supplementary file 1 — Tables [file ACM2-23-e13548-s002.docx]

**Supplementary table S1:**

Table S1. Surface dose in % with minimum values shaded light grey and maximum values shaded dark grey. Single standard deviation is computed.

| **6 MV, 0^o^** |  | **1 mm** | **2 mm** | **3 mm** | **4 mm** | **5 mm** |
| --- | --- | --- | --- | --- | --- | --- |
|  | Black | 40 ± 2 | 53 ± 1 | 62 ± 1 | 71 ± 1 | 73 ± 2 |
|  | Cyan | 38 ± 1 | 52 ± 1 | 62 ± 1 | 69 ± 2 | 76 ± 1 |
|  | Yellow | 41 ± 2 | 53 ± 1 | 67 ± 1 | 71 ± 1 | 76 ± 1 |
|  | Magenta | 41 ± 1 | 53 ± 2 | 62 ± 1 | 70 ± 2 | 76 ± 1 |
|  | White | 42 ± 2 | 53 ± 1 | 61 ± 1 | 70 ± 1 | 84 ± 1 |
| **18 MV, 0^o^** | Black | Not measured | 29 ± 2 | 37 ± 1 | 43 ± 1 | 50 ± 2 |
|  | Cyan |  | 34 ± 1 | 40 ± 1 | 47 ± 1 | 54 ± 1 |
|  | Yellow |  | 31 ± 2 | 40 ± 2 | 44 ± 1 | 50 ± 2 |
|  | Magenta |  | 33 ± 1 | 41 ± 2 | 47 ± 1 | 52 ± 1 |
|  | White |  | 32 ± 1 | 40 ± 1 | 46 ± 1 | 53 ± 1 |
| **6 MV, white** | 0^o^ | 42 ± 2 | 53 ± 1 | 61 ± 1 | 70 ± 1 | 84 ± 1 |
|  | 45^o^ | 45 ± 1 | 60 ± 1 | 70 ± 2 | 76 ± 1 | 82 ± 1 |
|  | 90^o^ | 44 ± 1 | 56 ± 1 | 70 ± 1 | 77 ± 1 | 82 ± 1 |
| **Print reproducibility**  **6 MV, 3 mm** | White |  |  | 61 ± 1 |  |  |
|  |  |  |  | 60 ± 1 |  |  |
|  |  |  |  | 64 ± 2 |  |  |
|  |  |  |  | 65 ± 1 |  |  |
|  |  |  |  | 64 ± 2 |  |  |

**Supplementary table S2:**

Table S2. Computing the density of samples with different colours and different print orientations, printed in three different days

| **Print Specs** | | | | **Measurements** | | | | | |
| --- | --- | --- | --- | --- | --- | --- | --- | --- | --- |
| **Print day** | **Colour** | **Print orientation (^o^)** | **Dimensions (mm)** | **Weight (g)** | **Thickness (mm)** | **Length (mm)** | **Width (mm)** | **Density (g/cm^3^)** | **Hounsfield Unit (HU)** |
| **Day 1** | Black | 0 | 30×30×5 | 3.81 | 4.97 | 30.25 | 30.15 | 0.840535 | -45 |
|  | Black | 20 | 30×30×5 | 4.048 | 5.04 | 30.33 | 30.15 | 0.878315 | 14 |
|  | Black | 45 | 30×30×5 | 4.08 | 5.11 | 30.38 | 30.26 | 0.868525 | 5 |
|  | Black | 70 | 30×30×5 | 4.40 | 5.29 | 30.28 | 30.35 | 0.905071 | -1 |
|  | Black | 90 | 30×30×5 | 4.19 | 5.21 | 30.2 | 30.44 | 0.874832 | 2 |
|  | White | 0 | 30×30×5 | 3.70 | 4.86 | 29.98 | 29.89 | 0.849587 | -21 |
|  | Yellow | 0 | 30×30×5 | 4.03 | 4.87 | 30.29 | 30.26 | 0.902834 | 2 |
|  | Magenta | 0 | 30×30×5 | 3.80 | 4.90 | 30.01 | 30.16 | 0.856821 | -54 |
|  | Cyan | 0 | 30×30×5 | 3.54 | 4.93 | 30.10 | 30.01 | 0.794921 | -86 |
| **Day 2** | Black | 0 | 30×30×5 | 3.74 | 5.03 | 30.15 | 30.11 | 0.819041 | -102 |
|  | Black | 20 | 30×30×5 | 4.27 | 5.12 | 30.34 | 30.32 | 0.906595 | -16 |
|  | Black | 45 | 30×30×5 | 4.23 | 5.17 | 30.30 | 30.19 | 0.894425 | -20 |
|  | Black | 70 | 30×30×5 | 4.27 | 5.20 | 30.24 | 30.31 | 0.895894 | 23 |
|  | Black | 90 | 30×30×5 | 4.10 | 5.25 | 30.12 | 30.16 | 0.859683 | 9 |
|  | White | 0 | 30×30×5 | 3.99 | 4.86 | 30.31 | 30.19 | 0.897197 | -69 |
|  | Yellow | 0 | 30×30×5 | 3.94 | 4.85 | 30.25 | 30.13 | 0.891312 | 16 |
|  | Magenta | 0 | 30×30×5 | 3.74 | 4.95 | 30.12 | 30.18 | 0.831174 | -90 |
|  | Cyan | 0 | 30×30×5 | 3.77 | 4.99 | 30.16 | 30.09 | 0.832506 | -94 |
| **Day 3** | Black | 0 | 30×30×5 | 3.78 | 5.03 | 30.08 | 30.25 | 0.825887 | -89 |
|  | Black | 20 | 30×30×5 | 4.29 | 5.13 | 30.40 | 30.42 | 0.904289 | 1 |
|  | Black | 45 | 30×30×5 | 4.24 | 5.16 | 30.34 | 30.28 | 0.894427 | 11 |
|  | Black | 70 | 30×30×5 | 4.31 | 5.25 | 30.43 | 30.35 | 0.888909 | 17 |
|  | Black | 90 | 30×30×5 | 4.15 | 5.30 | 30.18 | 30.20 | 0.859105 | 11 |
|  | White | 0 | 30×30×5 | 4.02 | 4.86 | 30.18 | 30.20 | 0.907535 | -31 |
|  | Yellow | 0 | 30×30×5 | 3.98 | 4.85 | 30.18 | 30.10 | 0.903349 | -43 |
|  | Magenta | 0 | 30×30×5 | 3.79 | 4.95 | 30.23 | 30.04 | 0.843133 | -141 |
|  | Cyan | 0 | 30×30×5 | 3.76 | 4.95 | 30.24 | 30.11 | 0.834238 | -75 |

**Supplementary table S3:**

Table S3. Ultimate tensile strength of 35 samples with different print orientations, thicknesses, and colours is computed. Standard deviation is computed for type (A) uncertainty.

| **Samples** | | **Ultimate Tensile Strength (MPa)** |
| --- | --- | --- |
| **Orientation and Colour** | **Thickness** |  |
| 0^o^-Black | 1 mm | 15.6±0.5 |
|  | 2 mm | 22±0.1 |
|  | 3 mm | 24.8±0.2 |
|  | 4 mm | 27.9±0.8 |
|  | 5 mm | 31.5±0.9 |
| 0^o^-Cyan | 1 mm | 19.6±1 |
|  | 2 mm | 29.4±0.4 |
|  | 3 mm | 32.3±0.3 |
|  | 4 mm | 33.1±0.5 |
|  | 5 mm | 36±0.6 |
| 0^o^-Magenta | 1 mm | 23.3±0.8 |
|  | 2 mm | 30.8±1.6 |
|  | 3 mm | 32.4±1.1 |
|  | 4 mm | 35.4±0.6 |
|  | 5 mm | 37.3±1.5 |
| 0^o^-Yellow | 1 mm | 22.6±0.5 |
|  | 2 mm | 28±0.6 |
|  | 3 mm | 31.5±0.6 |
|  | 4 mm | 34±0.6 |
|  | 5 mm | 38.1±0.7 |
| 0^o^-White | 1 mm | 23.4±0.7 |
|  | 2 mm | 29.5±0.3 |
|  | 3 mm | 32.1±0.9 |
|  | 4 mm | 34.2±0.6 |
|  | 5 mm | 37.9±1.9 |
| 45^o^-White | 1 mm | 18±0.3 |
|  | 2 mm | 29.9±1.2 |
|  | 3 mm | 36.6±0.4 |
|  | 4 mm | 37.7±0.3 |
|  | 5 mm | 40±0.7 |
| 90^o^-White | 1 mm | 17±0.7 |
|  | 2 mm | 26.4±0.5 |
|  | 3 mm | 32.9±0.9 |
|  | 4 mm | 33.5±0.9 |
|  | 5 mm | 37±0.6 |
