# Supplementary material for: Multi‐jet fusion for additive manufacturing of radiotherapy immobilization devices: Effects of color, thickness, and orientation on surface dose and tensile strength
Source: J Appl Clin Med Phys. 2022 Feb 25;23(4):e13548. doi: 10.1002/acm2.13548 (PMC8992947; doi:10.1002/acm2.13548)
Supplement: Supplementary file 2 — Supporting Information [file ACM2-23-e13548-s001.docx]

**Multi Jet Fusion for Additive Manufacturing of Radiotherapy Immobilization Devices: Effects of Colour, Thickness and Orientation on Surface Dose and Tensile Strength**

**Amirhossein Asfia**

1. School of Engineering

Faculty of Science, Engineering and Built Environment

Deakin University

75 Pigdons Road,

Waurn Ponds, VIC, 3216, Australia

2. ARC Industrial Transformation Training Centre in Additive Bio-manufacturing

Queensland University of Technology

60 Musk Avenue

Kelvin Grove, QLD, 4059, Australia

**Email address:** asfiaamirhosein@gmail.com*

**Basaula Deepak**

1. Peter MacCallum Cancer Centre

Department of Physical Science

305 Grattan Street

Parkville, VIC, 3000, Australia

**Email address:** [Deepak.Basaula@petermac.org](mailto:Deepak.Basaula@petermac.org)

**James I. Novak**

1. School of Architecture

Faculty of Engineering, Architecture and Information Technology

The University of Queensland

St Lucia, QLD, 4072, Australia

2. Herston Biofabrication Institute

Metro North Hospital and Health Service

Level 12, Block 7, Royal Brisbane and Women’s Hospital,

Herston, QLD, 4029, Australia

**Email address:** james.novak@health.qld.gov.au

**Bernard Rolfe**

1. School of Engineering

Faculty of Science, Engineering and Built Environment

Deakin University

75 Pigdons Road,

Waurn Ponds, VIC, 3216, Australia

**Email address:** [Bernard.rolfe@deakin.edu.au](mailto:Bernard.rolfe@deakin.edu.au)

**Tomas Kron**

1. Peter MacCallum Cancer Centre

Department of Physical Science

305 Grattan Street

Parkville, VIC, 3000, Australia

2. ARC Industrial Transformation Training Centre in Additive Bio-manufacturing

Queensland University of Technology

60 Musk Avenue

Kelvin Grove, QLD, 4059, Australia

**Email address:** [Tomas.Kron@petermac.org](mailto:Tomas.Kron@petermac.org)

**Author to whom correspondence should be addressed:**

**Name:** Amirhossein Asfia

**Address:** 75 Pigdons road, Waurn Ponds, Vic, 3216, Australia

**E-mail:** asfiaamirhosein@gmail.com

**Running title:** Effect of colour, print orientation, and thickness in making immobilization devices using MJF technology

**Author contribution statement:**

Amirhossein Asfia performed the tensile strength and skin dose measurement experiments, analysed the data and wrote the manuscript. Basaula Deepak performed the skin dose measurement test and analysed the data. James Novak, Bernard Rolfe, and Tomas Kron supervised the work, analysed the data, designed the experiments and revised the manuscript. All authors revised the manuscript.
